# Supplementary figures and images for: Medical management of muscle weakness in Duchenne muscular dystrophy
Source: PLoS One. 2020 Oct 19;15(10):e0240687. doi: 10.1371/journal.pone.0240687 (PMC7571693; doi:10.1371/journal.pone.0240687)

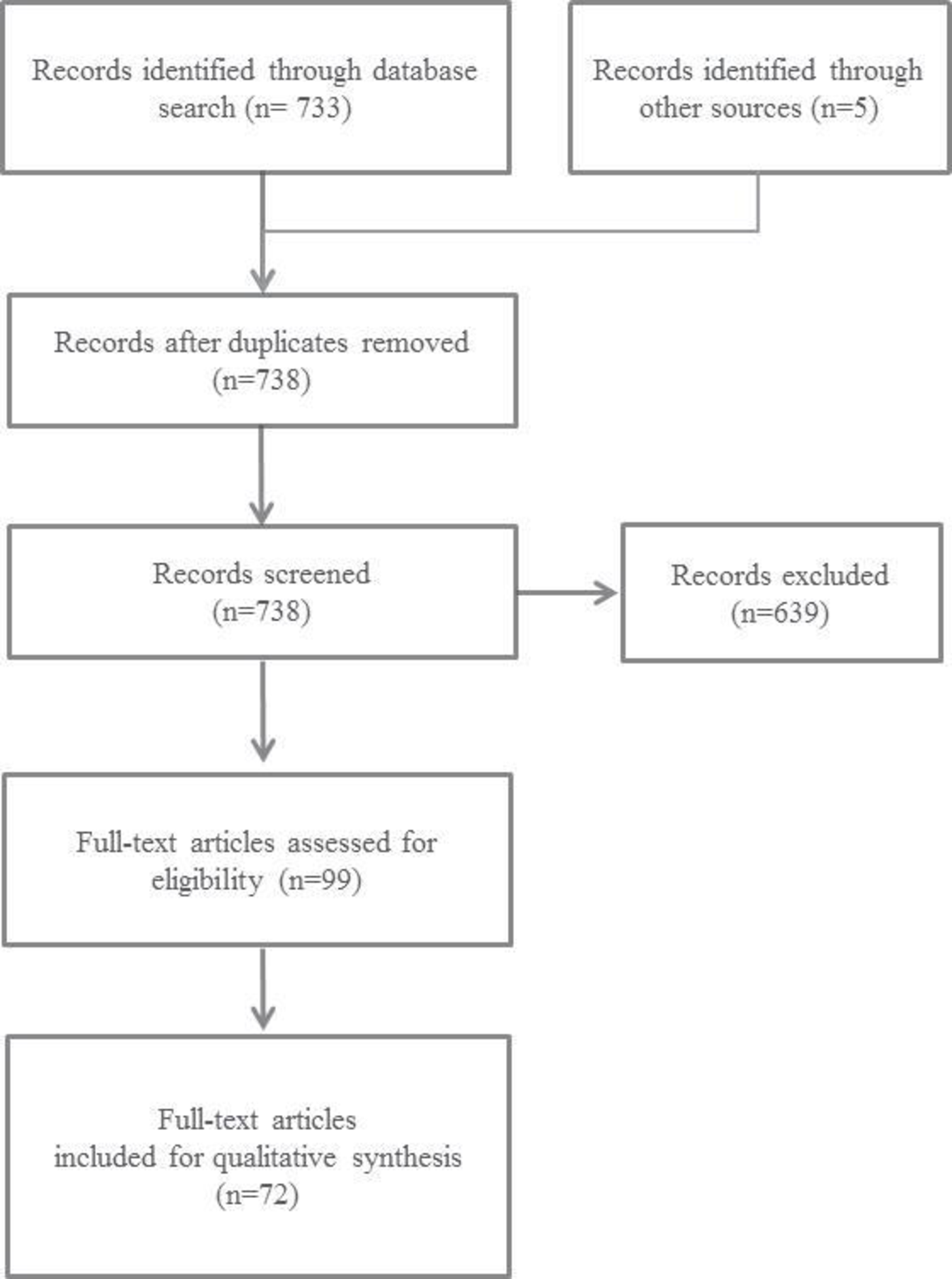

Supplement: S1 Fig — (TIF) [file pone.0240687.s001.tif]
