# Supplementary material for: Medical management of muscle weakness in Duchenne muscular dystrophy
Source: PLoS One. 2020 Oct 19;15(10):e0240687. doi: 10.1371/journal.pone.0240687 (PMC7571693; doi:10.1371/journal.pone.0240687)
Supplement: S1 Table — (DOCX) [file pone.0240687.s002.docx]

S1 Table. Consensus statements & level of agreement (Survey round 1)

| **Consensus statements (Round 1)** | **Strongly Agree** | **Agree** | **Neither agree nor disagree** | **Disagree** | **Strongly disagree** |
| --- | --- | --- | --- | --- | --- |
| **Statement 1:** Corticosteroid Treatment |  |  |  |  |  |
| a.       Corticosteroids are the preferred treatment option to slow the progression of muscle weakness and delay complications of the disease. | 11 | 4 | 0 | 0 | 0 |
| b.       Corticosteroids can increase ambulation by 1–2 years. | 10 | 5 | 0 | 0 | 0 |
| **Statement 2:** Corticosteroid Safety |  |  |  |  |  |
| a.       Approximately 50% of patients discontinue daily corticosteroids therapy due to side effects. | 1 | 1 | 1 | 10 | 2 |
| b.       Before starting corticosteroids adverse effects (such as obesity, delayed puberty, adrenal crisis, behavioral disturbance, growth retardation, osteoporosis, hypertension, and cataract) must be explained to patients/caregiver. | 15 | 0 | 0 | 0 | 0 |
| **Statement 3:** Daily deflazacort is reported to demonstrate similar benefits compared to daily prednisone with different side effects. | 2 | 11 | 1 | 1 | 0 |
| **Statement 4:** Daily prednisone (DP) vs Daily deflazacort (DD) has been studied in a randomized trial over one year. Do you believe: |  |  |  |  |  |
| a.       DP is more effective that DD | 0 | 0 | 5 | 7 | 3 |
| b.       DP reduces time to loss of ambulation when compared to DD | 0 | 3 | 5 | 6 | 1 |
| c.       DP is more likely to lead to obesity than DD | 0 | 10 | 3 | 2 | 0 |
| d.       DP is more likely to cause delays in puberty than DD | 0 | 0 | 8 | 6 | 1 |
| e.       DP is more likely to cause an adrenal crisis than DD | 0 | 0 | 6 | 8 | 1 |
| [f.        Behavioral disturbances are more common with DP than DD](https://www.ncbi.nlm.nih.gov/pubmed/27525172) | 2 | 5 | 5 | 3 | 0 |
| g.       DP is more likely to cause osteoporosis than DD | 0 | 0 | 8 | 6 | 1 |
| h.       DP is more likely to cause hypertension than DD | 0 | 0 | 9 | 5 | 1 |
| i.        DP is more likely to cause cataracts than DD | 0 | 0 | 1 | 9 | 5 |
| **Statement 5**: Daily deflazacort (DD) vs Weekend prednisone (WP) has never been studied head to head. Do you believe |  |  |  |  |  |
| a.       DD is more effective than WP | 1 | 3 | 7 | 4 | 0 |
| b.       DD reduces time to loss of ambulation when compared to WP | 1 | 2 | 9 | 3 | 0 |
| c.       DD is more likely to lead to obesity than WP | 2 | 7 | 4 | 2 | 0 |
| d.       DD is more likely to cause delays in puberty than WP | 4 | 6 | 5 | 0 | 0 |
| e.       DD is more likely to cause an adrenal crisis than WP | 4 | 7 | 3 | 1 | 0 |
| f.        Behavioral disturbances are more common with DD than WP | 1 | 4 | 8 | 2 | 0 |
| g.       DD is more likely to cause osteoporosis than WP | 3 | 6 | 5 | 1 | 0 |
| h.       DD is more likely to cause hypertension than WP | 3 | 2 | 10 | 0 | 0 |
| i.        DD is more likely to cause cataracts than WP | 7 | 7 | 1 | 0 | 0 |
| **Statement 6:** Strength of Evidence |  |  |  |  |  |
| a.       There is strong evidence that daily use of prednisone is beneficial. | 11 | 4 | 0 | 0 | 0 |
| b.       There is strong evidence that weekend use of prednisone is beneficial. | 4 | 9 | 0 | 2 | 0 |
| c.       There is strong evidence that daily use of deflazacort is beneficial. | 10 | 5 | 0 | 0 | 0 |
| **Statement 7:** Please rank the following steroid treatments based on your preference. Rankings can be equal if there is no preference for one over the other based on the ages presented below. |  |  |  |  |  |
| AGE: Less than age 3 | **DP** |  | **WP** |  | **DD** |
| -    Rank 1 (no. of response) | 0 |  | 12 |  | 1 |
| -    Rank 2 (no. of response) | 5 |  | 0 |  | 9 |
| -    Rank 3 (no. of response) | 8 |  | 1 |  | 3 |
| AGE 3-6 | **DP** |  | **WP** |  | **DD** |
| -    Rank 1 (no. of response) | 2 |  | 9 |  | 5 |
| -    Rank 2 (no. of response) | 8 |  | 1 |  | 8 |
| -    Rank 3 (no. of response) | 5 |  | 5 |  | 2 |
| Age >6 years | **DP** |  | **WP** |  | **DD** |
| -    Rank 1 (no. of response) | 3 |  | 7 |  | 7 |
| -    Rank 2 (no. of response) | 7 |  | 2 |  | 6 |
| -    Rank 3 (no. of response) | 5 |  | 6 |  | 2 |
| **Statement 8:** Out of pocket cost to the family should be explored when considering a steroid therapy. | 10 | 4 | 1 | 0 | 0 |
| **Statement 9:** Family Input |  |  |  |  |  |
| a.       It is acceptable for a family with a child >4 year to choose daily prednisone as the first steroid regimen. | 5 | 9 | 0 | 0 | 1 |
| b.       It is acceptable for a family with a child >4 years to choose weekend prednisone as the first steroid regimen. | 6 | 7 | 2 | 0 | 0 |
| c.       It is acceptable for a family with a child >4 years to choose deflazacort as the first steroid regimen. | 6 | 5 | 3 | 0 | 1 |
| **Statement 10:** Eteplirsen should be offered to patients with confirmed mutation of the DMD gene amenable to exon 51 skipping. | 9 | 6 | 0 | 0 | 0 |
| **Statement 11:** Eteplirsen effectiveness |  |  |  |  |  |
| a.       Eteplirsen use is associated with delay in time to loss of ambulation and disease milestones | 1 | 10 | 4 | 0 | 0 |
| b.       Eteplirsen is likely to slow disease progression | 2 | 12 | 1 | 0 | 0 |
| **Statement 12:** Eteplirsen Use in children with exon51 skippable mutations |  |  |  |  |  |
| a.       Eteplirsen should be offered at diagnosis | 6 | 9 | 0 | 0 | 0 |
| b.       Eteplirsen should be offered when a patient can no longer rise from floor but can walk | 4 | 7 | 2 | 1 | 1 |
| c.       Eteplirsen should be offered when a patient has recently lost ambulation | 3 | 8 | 3 | 0 | 1 |
| d.       Eteplirsen should be offered when a patient can still feed but cannot wash their hair | 3 | 8 | 3 | 1 | 0 |
| e.       Eteplirsen should be offered when a patient is on fulltime ventilation and cannot power a wheelchair independently. | 0 | 0 | 3 | 11 | 1 |
| f.        Eteplirsen should not be offered. | 0 | 0 | 0 | 5 | 10 |
| **Statement 13:** Measuring Effectiveness |  |  |  |  |  |
| a.       Muscle biopsy should be done to measure the effectiveness of Eteplirsen | 0 | 0 | 0 | 3 | 12 |
| b.       Clinical Measures (motor outcomes, motor milestones, pulmonary function tests) are an appropriate and sufficient way to measure the effectiveness of eteplirsen. | 3 | 10 | 1 | 0 | 1 |
| **Statement 14:** Eteplirsen place in therapy |  |  |  |  |  |
| a. Eteplirsen and steroid therapy are complementary treatments. | 9 | 6 | 0 | 0 | 0 |
| b.       Eteplirsen and steroid therapy can be used together | 11 | 4 | 0 | 0 | 0 |
| **Statement 15:** Long term treatment with eteplirsen may slow the respiratory decline and deterioration of cardiac function in patients with DMD | 2 | 8 | 4 | 1 | 0 |

**Abbreviations:** DD: daily deflazacort; DP: daily prednisone; WP: weekend prednisone
