# Supplementary material for: Medical management of muscle weakness in Duchenne muscular dystrophy
Source: PLoS One. 2020 Oct 19;15(10):e0240687. doi: 10.1371/journal.pone.0240687 (PMC7571693; doi:10.1371/journal.pone.0240687)
Supplement: S2 Table — (DOCX) [file pone.0240687.s003.docx]

S2 Table. Consensus statements & level of agreement for treatment with corticosteroids (Survey round 2&4)

| **Consensus statements (Round 2&4)** | **Strongly Agree** | **Agree** | **Neither agree nor disagree** | **Disagree** | **Strongly disagree** |
| --- | --- | --- | --- | --- | --- |
| **Statement 1: Corticosteroid Treatment** |  |  |  |  |  |
| a.       Corticosteroids are the preferred treatment option to slow the progression of muscle weakness and delay complications of the disease. | 11 | 4 | 0 | 0 | 0 |
| b.       Corticosteroids can increase ambulation by 1–3 years. | 11 | 4 | 0 | 0 | 0 |
| **Statement 2: Corticosteroid Safety: It is reported in studies that patients discontinue or do not start steroid treatment due to side effects** |  |  |  |  |  |
| a.    Concern about side effects play a major role in a starting or forgoing steroid treatment. | 9 | 5 | 0 | 1 | 0 |
| b.       Side effects play major role in steroid dosing adjustments. | 9 | 6 | 0 | 0 | 0 |
| c.       Before starting corticosteroids adverse effects must be explained to patients/caregiver. | 15 | 0 | 0 | 0 | 0 |
| **Statement 3: Daily deflazacort is reported to demonstrate similar benefits compared to daily prednisone with different side effects.** |  |  |  |  |  |
| a.       DD and DP are both effective in the treatment of DMD. | 10 | 4 | 0 | 1 | 0 |
| b.       DD and DP have different side effects. | 2 | 10 | 2 | 1 | 0 |
| **Statement 4: Daily prednisone (DP) vs Weekend Prednisone (WP) has been studied in a randomized trial over one year. Do you believe over a longer period of time:** |  |  |  |  |  |
| a.       DP is more effective that WP | 0 | 4 | 9 | 2 | 0 |
| b.       DP reduces time to loss of ambulation when compared to WP | 0 | 1 | 10 | 4 | 0 |
| c.       DP is more likely to lead to obesity than WP | 3 | 11 | 1 | 0 | 0 |
| d.       DP is more likely to cause delays in puberty than WP | 5 | 7 | 3 | 0 | 0 |
| e.       DP is more likely to cause an adrenal crisis than WP, if stopped abruptly | 4 | 7 | 4 | 0 | 0 |
| f.        Behavioral disturbances are more common with DP than WP | 1 | 10 | 3 | 1 | 0 |
| g.       DP is more likely to cause osteoporosis than WP | 3 | 6 | 6 | 0 | 0 |
| h.       DP is more likely to cause hypertension than WP | 2 | 4 | 8 | 1 | 0 |
| i.        DP is more likely to cause cataracts than WP | 3 | 4 | 8 | 0 | 0 |
| **Statement 5: Daily prednisone (DP) vs Daily deflazacort (DD) has been studied in a randomized trial over one year. Do you believe over a longer period of time:** |  |  |  |  |  |
| a.       DP is more effective that DD | 0 | 0 | 5 | 8 | 2 |
| b.       DP reduces time to loss of ambulation when compared to DD | 0 | 3 | 4 | 8 | 0 |
| c.       DP is more likely to lead to obesity than DD | 3 | 7 | 3 | 2 | 0 |
| d.       DP is more likely to cause delays in puberty than DD | 0 | 0 | 8 | 7 | 0 |
| e.       DP is more likely to cause an adrenal crisis than DD, if stopped abruptly | 0 | 0 | 8 | 6 | 1 |
| f.        Behavioral disturbances are more common with DP than DD | 0 | 8 | 5 | 2 | 0 |
| g.       DP is more likely to cause osteoporosis than DD | 0 | 1 | 7 | 7 | 0 |
| h.       DP is more likely to cause hypertension than DD | 0 | 0 | 8 | 7 | 0 |
| i.        DP is more likely to cause cataracts than DD | 0 | 1 | 0 | 7 | 7 |
| **Statement 6: Daily deflazacort (DD) vs Weekend prednisone (WP) has never been studied head to head. Do you believe:** |  |  |  |  |  |
| a.       DD is more effective than WP | 0 | 6 | 6 | 2 | 1 |
| b.       DD reduces time to loss of ambulation when compared to WP | 0 | 0 | 11 | 2 | 2 |
| c.       DD is more likely to lead to obesity than WP | 1 | 7 | 6 | 0 | 1 |
| d.       DD is more likely to cause delays in puberty than WP | 2 | 6 | 6 | 1 | 0 |
| e.       DD is more likely to cause an adrenal crisis than WP, if stopped abruptly | 3 | 9 | 3 | 0 | 0 |
| f.        Behavioral disturbances are more common with DD than WP. | 0 | 4 | 8 | 3 | 0 |
| g.       DD is more likely to cause osteoporosis than WP | 2 | 4 | 9 | 0 | 0 |
| h.       DD is more likely to cause hypertension than WP | 2 | 4 | 9 | 0 | 0 |
| i.        DD is more likely to cause cataracts than WP | 5 | 10 | 0 | 0 | 0 |
| **Statement 7: Strength of Evidence** |  |  |  |  |  |
| a.       There is strong evidence that daily use of prednisone is beneficial. | 10 | 5 | 0 | 0 | 0 |
| b.       There is strong evidence that weekend use of prednisone is beneficial. | 3 | 9 | 2 | 1 | 0 |
| c.       There is strong evidence that daily use of deflazacort is beneficial. | 10 | 5 | 0 | 0 | 0 |
| **Statement 8: Cost Impact** |  |  |  |  |  |
| a. Out of pocket cost to the family should be explored when considering a steroid therapy. | 8 | 6 | 0 | 1 | 0 |
| **Statement 9: Family Input** |  |  |  |  |  |
| a.       For patients where steroid use is clinically appropriate, it is acceptable for a family to choose daily prednisone as the first steroid regimen. | 4 | 10 | 1 | 0 | 0 |
| b.      For patients where steroid use is clinically appropriate, it is acceptable for a family to choose weekend prednisone as the first steroid regimen. | 8 | 5 | 1 | 1 | 0 |
| c.       For patients where steroid use is clinically appropriate, it is acceptable for a family to choose deflazacort as the first steroid regimen. | 6 | 7 | 1 | 1 | 0 |
| **Statement 10: Comparative effectiveness** | **Yes** | **No** | **Uncertain** |  |  |
| a.       Do you believe daily prednisone and daily deflazacort have equal efficacy? | 8 | 5 | 2 |  |  |
| b.       Do you believe daily prednisone and weekend prednisone have equal efficacy? | 6 | 4 | 5 |  |  |
| c.       Do you believe weekend prednisone and daily deflazacort have equal efficacy? | 4 | 5 | 6 |  |  |

**Abbreviations:** DD: daily deflazacort; DP: daily prednisone; WP: weekend prednisone
