# Supplementary material for: Medical management of muscle weakness in Duchenne muscular dystrophy
Source: PLoS One. 2020 Oct 19;15(10):e0240687. doi: 10.1371/journal.pone.0240687 (PMC7571693; doi:10.1371/journal.pone.0240687)
Supplement: S3 Table — (DOCX) [file pone.0240687.s004.docx]

S3 Table: Consensus statements & level of agreement for treatment with eteplirsen (Survey round 2&4)

| **Consensus statements (Round 2&4)** | **Strongly Agree** | **Agree** | **Neither agree nor disagree** | **Disagree** | **Strongly disagree** |
| --- | --- | --- | --- | --- | --- |
| **Statement 1: Using Eteplirsen** |  |  |  |  |  |
| a. Eteplirsen should be offered to patients with confirmed mutation of the *DMD* gene amenable to exon 51 skipping. | 10 | 5 | 0 | 0 | 0 |
| **Statement 2: Eteplirsen effectiveness** |  |  |  |  |  |
| a.       Eteplirsen use is associated with delay in time to loss of ambulation and disease milestones. | 5 | 7 | 3 | 0 | 0 |
| b.       Eteplirsen is likely to slow disease progression. | 4 | 9 | 2 | 0 | 0 |
| **Statement 3: Eteplirsen Place in Therapy** |  |  |  |  |  |
| a.       Eteplirsen and steroid therapy are complementary treatments and should be used concomitantly | 7 | 8 | 0 | 0 | 0 |
| b. Eteplirsen and steroid therapy can be used together | 12 | 3 | 0 | 0 | 0 |
| **Statement 4: For patients currently in your practice, at different stages of disease progression, do you believe:** |  |  |  |  |  |
| a. Eteplirsen should be offered at diagnosis | 11 | 4 | 0 | 0 | 0 |
| b. Eteplirsen should be offered to a patient who can no longer rise from floor but can walk | 9 | 5 | 1 | 0 | 0 |
| c. Eteplirsen should be offered to a patient who has recently lost ambulation | 6 | 7 | 1 | 1 | 0 |
| d. Eteplirsen should be offered to a patient who can still feed but cannot wash their hair | 5 | 7 | 1 | 2 | 0 |
| e. Eteplirsen should be offered to a patient who is on fulltime ventilation and cannot power a wheelchair independently. | 0 | 0 | 1 | 10 | 4 |
| f. Eteplirsen should not be offered at any stage of disease progression. | 0 | 1 | 0 | 4 | 10 |
| **Statement 5: Measuring Effectiveness** |  |  |  |  |  |
| a.       Muscle biopsy should be done to measure the effectiveness of Eteplirsen | 0 | 0 | 1 | 3 | 11 |
| b.       Clinical Measures (motor outcomes, motor milestones, pulmonary function tests) are an appropriate and sufficient way to measure the effectiveness of eteplirsen. | 4 | 9 | 1 | 1 | 0 |
| **Statement 6: Benefit of Eteplirsen** |  |  |  |  |  |
| a.       Long term use of eteplirsen delays respiratory decline in patients with DM | 2 | 10 | 2 | 1 | 0 |
| b.       Long term use of eteplirsen slows deterioration of cardiac function in patients with DMD | 0 | 3 | 8 | 4 | 0 |
| **Statement 7: Eteplirsen Burden and Safety** |  |  |  |  |  |
| a.       The impact of weekly infusion should be discussed prior to starting eteplirsen | 11 | 4 | 0 | 0 | 0 |
| b.       Before starting eteplirsen adverse effects must be explained to patients/caregiver. | 14 | 1 | 0 | 0 | 0 |
| c.       Outside of problems associated with weekly infusions, eteplirsen is well tolerated. | 8 | 7 | 0 | 0 | 0 |
| **Statement 8: Treatment Initiation** |  |  |  |  |  |
| For young patients with exon 51 skip-amenable mutations not yet ready to begin corticosteroid treatment, eteplirsen should be offered. | 9 | 5 | 1 | 0 | 0 |
| **Statement 9: Effectiveness Measures** |  |  |  |  |  |
| a.       Clinical measures (motor outcomes, motor milestones, pulmonary function tests) allow you to monitor disease progression. | 12 | 3 | 0 | 0 | 0 |
| b.       Corticosteroids and eteplirsen are proposed to slow disease progression; however, it is not possible in the clinical setting in an individual patient to reliably measure this slowing of the disease progression. | 1 | 4 | 1 | 8 | 1 |
